# Forty-Year Follow-up of Full-Thickness Skin Graft After Thermal Burn Injury to the Volar Hand

**Published:** 2016-07-28

**Authors:** Dexter Weeks, Morton L. Kasdan, Bradon J. Wilhelmi

**Affiliations:** ^a^University of Louisville School of Medicine, Louisville, Ky; ^b^Robley Rex Veterans Affairs Medical Center, Louisville, Ky; ^c^Division of Plastic Surgery, Department of Surgery, University of Louisville, Louisville, Ky

**Keywords:** volar hand burn, burn reconstruction, burn injury reconstruction, full-thickness skin graft, thermal burn injury

## Abstract

**Background:** The hands are commonly affected in severe thermal burn injuries. Resulting contractures lead to significant loss of function. Burn contracture release and skin grafting are necessary to restore hand function. We report a case in which surgical reconstruction of a volar hand burn was performed with full-thickness skin grafting. The patient had a 40-year follow-up to assess the function and cosmesis of the repaired hand. **Methods:** We report a case in which a 15-month-old boy presented after receiving third-degree burns to the left volar hand, including the flexural aspects of the index, long, and ring fingers by placing it on a hot kitchen stove burner. The patient subsequently underwent scar contracture release and full-thickness skin grafting. **Results:** Eleven years after reconstruction, further contractures developed associated with the patient's growth, which were reconstructed with repeat full-thickness skin graft from the inguinal region. No recurrence was witnessed afterward and 40 years after initial injury, the patient maintains full activities of daily living and use of his hand in his occupation. **Conclusions:** There is debate regarding the superiority of split-thickness versus full-thickness grafts during reconstruction. Our case strengthens the argument for durability of a full-thickness skin graft following thermal burn injury.

Functional loss following burn injuries to the hand can be devastating. It has been reported that the hands are injured in more than 80% of all severe burns.^1^ A specific goal to surgical intervention in hand burns includes preserving tactile sensation, normal function, and restoration of appearance. Contractures of the hand are common following burns. The management of postburn contractures includes scar tissue release and skin grafting. Prior studies have investigated short-term outcomes following burn injuries to the hand. Few have investigated outcomes of pediatric hand burn injuries into later adulthood. In this case, we present a follow-up 40 years after burn scar contracture release with full-thickness skin grafts.

## CASE REPORT

**Figure 1:**
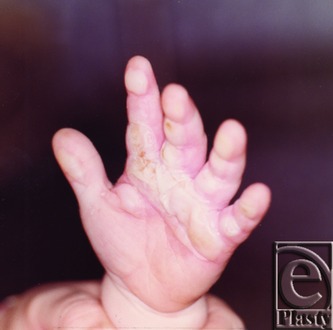
Burn injuries to flexural areas of the left volar hand in January 1976.

Our patient presented in January 1976 as an otherwise healthy 15-month-old child with third-degree burns to the volar hand, including the left index, long, and ring fingers that occurred 7 days before the consultation. The patient originally sustained third-degree burns to the hand after opening the oven door, climbing up, and placing the hand on a hot stovetop burner (Fig 1). At the time of presentation, there were no systemic sequelae of the burn and the blisters were intact. Acute management involved topical Silvadene application to the burned area. The patient was scheduled to follow-up with the surgeon regularly to assess need for future surgery. In May 1976, contractures of the left index, long, and ring fingers had developed and surgery was scheduled.

**Figure 2:**
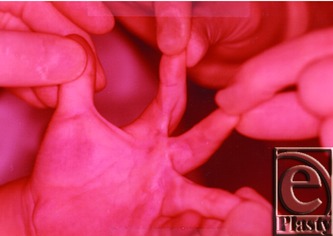
Photograph of the hand after follow-up in October 1976 showing scar maturation.

The patient was reexamined in October 1976 (Fig 2).

Transverse incisions were made to release the contractures and protect the neurovascular bundles on the left index, long, and ring fingers. It was noted that the dense scar caused shortening of the flexor tendon sheath of the same fingers. The defects were similar in size, but the contracture was most significant on the long finger. After the release, a full-thickness skin graft was taken from the left inguinal area. The graft took over the tendon and completely covered the defect. The patient was not cooperative at the dressing change 1 week later and required general anesthesia with ketamine.

**Figure 3:**
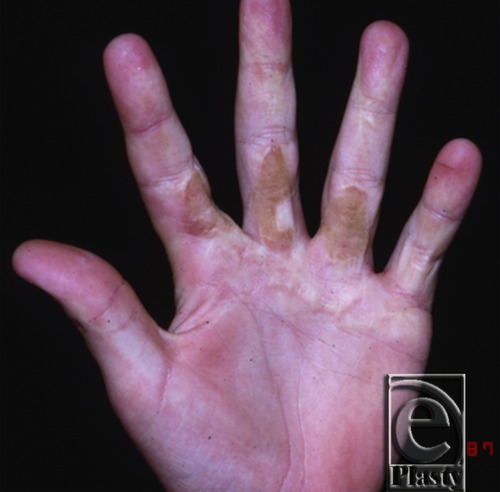
Hand in June 1987 with progressive contracture.

**Figure 4:**
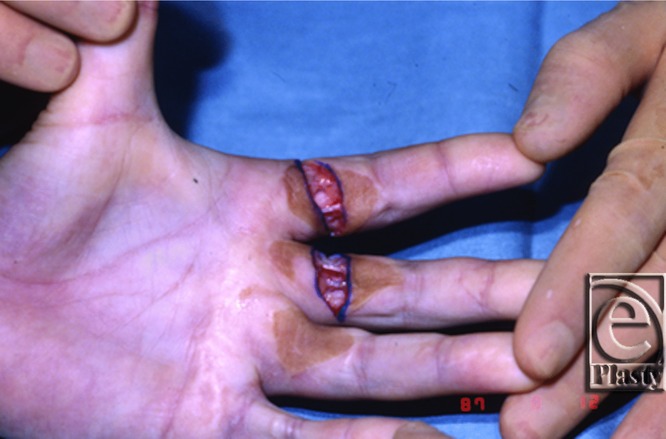
Hand at the time of surgery in August 1987.

The patient had excellent function until June 1987 (11 years later) when noted to develop progressive scar contracture at the age of 12 years (Fig 3). In August 1987 (Fig 4), the contractures were incised and a full-thickness skin graft was placed. There was extensive involvement around the neurovascular bundles, with the ulnar digital nerve of the index finger having the most involvement. Neurolysis of the neurovascular bundles of both digits and a repeat full-thickness skin graft from the right inguinal area were performed. The inguinal region was the donor site to avoid grafting skin bearing hair. Follow-up appointments after this surgery showed full range of motion and use for activities of daily living. There were no repeat incidents of contracture.

## RESULTS

**Figure 5:**
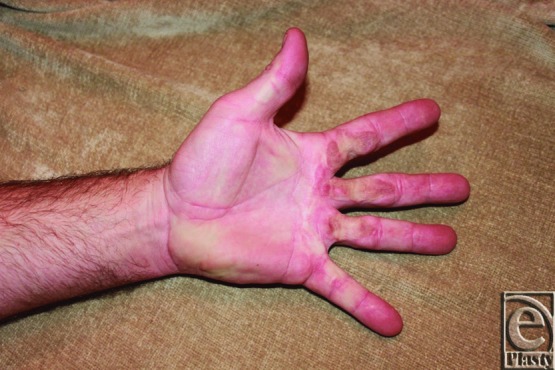
Patient's volar hand 40 years after initial burn injury.

**Figure 6:**
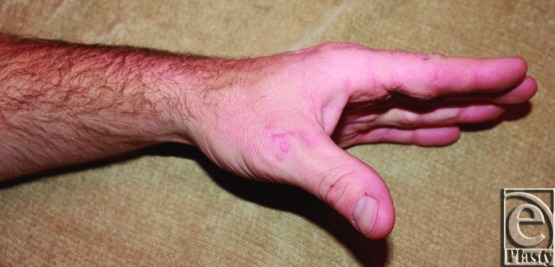
Patient's hand with full digital extension.

**Figure 7:**
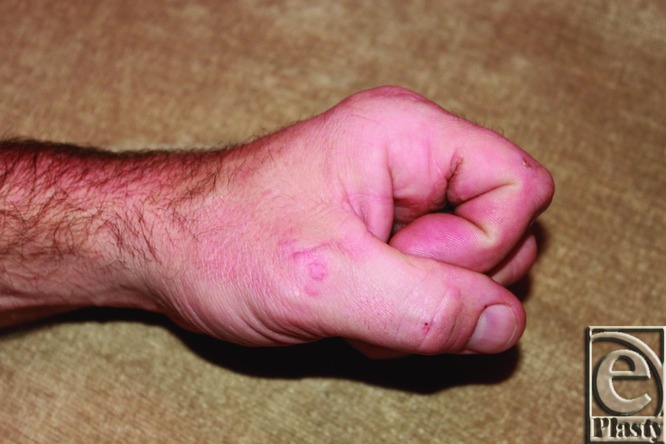
Patient's hand with digital flexion.

Forty years after initial burn injury, our patient has well-healed scars without any signs of repeat contracture (Fig 5). Functionally, his hand is unhindered for his activities of daily living. Current examination shows full range of motion for digital extension (Fig 6) and gripping (Fig 7). His current occupation requires heavy gripping and lifting.

## DISCUSSION

Current literature lacks consensus regarding superiority of full-thickness versus partial-thickness skin grafts for burn contracture reconstruction.^2^^-^4 The texture and color of volar hand skin are unique compared with other body skin and make donor selection difficult. Arguments for partial-thickness grafting suggest a decreased risk of palmar hyperpigmentation and less pain.^2^^-^4 Full-thickness grafts have decreased recurrence of contracture.^3^^,^^5^^,^^6^ These grafts also have increased capability for full flexion and extension of the hand and require less reoperation.^5^,^6^ The increased pliability prevents functional hand deficits compared with partial-thickness grafts.^7^

Prior studies established factors improving skin graft survivability up to 3 years.^8^-^10^ No study to date has followed a full-thickness skin graft for burn contracture reconstruction 40 years from the initial injury. The outcome strengthens arguments for the long-term durability of full-thickness skin grafting in hand burn injury. This case demonstrates that contracture release and soft-tissue reconstruction return adequate function to the hand even after significant thermal injury.
